# Differential expression profiling of long non-coding RNA GAS5 and miR-126-3p in human cancer cells in response to sorafenib

**DOI:** 10.1038/s41598-019-45604-2

**Published:** 2019-06-24

**Authors:** Teresa Faranda, Ilaria Grossi, Michele Manganelli, Eleonora Marchina, Gianluca Baiocchi, Nazario Portolani, Marialuisa Crosatti, Giuseppina De Petro, Alessandro Salvi

**Affiliations:** 1 Department of Molecular and Translational Medicine, Division of Biology and Genetics, University of Brescia, Brescia, Italy; 2 Department of Clinical and Experimental Sciences, Surgical Clinic, University of Brescia, Brescia, Italy; 3 Department of Respiratory Sciences, University of Leicester, Leicester, UK

**Keywords:** Tumour biomarkers, Long non-coding RNAs

## Abstract

Long non-coding RNAs (lncRNAs) and microRNAs are involved in numerous physio-pathological conditions included cancer. To better understand the molecular mechanism of the oral antitumor multikinase inhibitor sorafenib, we profiled the expression of a panel of lncRNAs and miRNAs by qPCR array in a sorafenib-treated hepatocellular carcinoma (HCC) cell line. Among the most affected ncRNAs, we found that sorafenib mediated the dysregulation of the lncRNAs GAS5, HOTTIP and HOXA-AS2 and the miR-126-3p, in a panel of human cancer cell lines (HCC, renal and breast carcinomas). By luciferase gene reporter assay, we discovered that GAS5 may act as a sponge for miR-126-3p in HCC cells. The expression level of GAS5 and miR-126-3p was verified in human liquid and/or solid biopsies from HCC patients. miR-126-3p expression in HCC tissues was decreased respect to their correspondent peritumoral tissues. The levels of plasmatic circulating miR-126-3p and GAS5 were significantly higher and lower in HCC patients compared to healthy subjects, respectively. This study highlighted the capability of sorafenib to modulate the expression of a wide range of ncRNAs and specifically, GAS5 and miR-126-3p were involved in the response to sorafenib of different cancer cell types.

## Introduction

Non-coding RNAs (ncRNAs) can be divided into 2 classes according to their size: short ncRNAs with less than 200 nt and long ncRNAs (lncRNAs) with more than 200 nt. lncRNAs are a heterogeneous group of RNAs ranging from 200 to 100,000 nt in length with very limited or absent protein-coding capacity. They have been implicated in controlling gene expression, imprinting, and inactivation of X-chromosome, but the effects or mechanisms of action of most lncRNAs remain unknown^1,2^. LncRNAs have been emerging as essential players in the pathogenesis of cancer and their dysregulation has been closely associated with tumor development, progression and metastasis^3,4^.

Among the short ncRNAs, microRNAs (miRNAs) are one of the best studied and characterized classes. Mature miRs are about 22 nt long and they mainly control post-transcriptional gene expression by promoting degradation or repressing translation of target mRNAs^5^. In human cancer, the dysregulation of miRNAs expression has been extensively reported^6,7^.

Human hepatocellular carcinoma (HCC) is the most common malignancy of the liver worldwide and ranks as the third cause of cancer-related deaths. HCC develops in cirrhotic liver in 80–90% of patients. HBV, HCV infections and alcohol abuse are the main risk factors of cirrhosis and consequently HCC^8,9^.

Sorafenib is an oral multikinase inhibitor used to treat advanced and unresecable HCCs^10^. It is a small molecule that inhibits several serine/threonine and tyrosine kinases (CRAF, BRAF, VEGFR-2 and -3, PDGFR-ß, FGFR-1, c-kit, and Fms-like tyrosine kinase 3 (Flt-3)) in multiple oncogenic signaling pathways^11,12^. Although it is an effective anti-tumor treatment, some patients are non-responder, that is resistant to or developing resistance during therapy with sorafenib. For these reasons, efforts have been put into identifying strategies to make sorafenib a more active drug and to discover biological biomarkers of sensitiveness or resistance^13^. It is known that sorafenib may exert its antitumor activity by a Ras/RAF/MEK/ERK signaling pathway independent-mechanism and that it can target unexpected molecules. In this context, sorafenib may mediate global mRNA expression changes^14^, modulate the miRNome^15^ and alter the global DNA methylation profile^16^. It has been demonstrated both *in vitro* and *in vivo* that ectopic modulation of lncRNAs and miRNAs may improve the effectiveness of sorafenib^17,18^.

The main aim of the present work was to study whether the treatment of HCC cells with sorafenib could lead to the dysregulation of the lncRNAs and miRNAs best characterized in physio-pathological conditions. The expression of the most dysregulated ncRNAs identified by qPCR-array was studied in tumor cells derived from renal cell carcinoma (RCC) and breast carcinoma in order to verify more global and wide effects of sorafenib in different cancer types. For RCC, the efficacy and safety of sorafenib has been proved and it is a therapeutic option to treat advanced RCC approved by FDA^19^. In breast cancer clinical trials, the efficacy of sorafenib in combinations with gemcitabine and/or capecitabine in locally advanced or metastatic disease is considered promising^20^. With the discovery of novel molecular biomarkers of response or resistance and new molecular therapeutic targets such as lncRNAs and miRs, it may be possible to identify new experimental strategies to improve the responsiveness of cancer cells to treatment.

## Materials and Methods

### Cell cultures and treatment with sorafenib

In this study, human tumor cell lines derived from hepatocellular (HA22T/VGH, HUH6, HepG2 and SKHep1C3), breast (MCF-7 and HCC 1937) and renal (ACHN, Caki-1 and CRBM 1990) carcinomas were used. The HA22T/VGH, HUH6, MCF-7 and HCC-1937 cell lines were maintained in RPMI-1640 (Life Technologies) with 100 nM Sodium Pyruvate (ThermoFisher Scientific). HepG2 and SKHep1Clone3 (SKHep1C3), selected from human HCC-derived cells (SKHep1: ATCC HTB-52), were maintained in Earle’s MEM (Life Technologies). The renal cancer cell lines ACHN, Caki-1 and CRBM-1990 were kindly provided by Dr Francesca Perut (Istituto Ortopedico Rizzoli, Bologna, Itay) and were maintained in Iscove’s Modified Dulbecco’s Medium (IMDM; Sigma-Aldrich). All culture media were supplemented with 10% Fetal Bovine Serum (Euroclone) and 10,000 U/ml penicillin/streptomycin (ThermoFisher Scientific). To generate sorafenib resistant cells, HA22T/VGH cells were treated with increasing concentration of sorafenib for about 6 months until the concentration of 10 μM sorafenib was reached.

Sorafenib was synthesized and provided by Bayer Corporation (West Haven, CT, USA). This compound was dissolved in 100% dimethyl sulfoxide (DMSO; Sigma-Aldrich) and diluted with RPMI-1640, MEM or IMDM to the required concentration. 0.1% DMSO was added to cultures as a solvent-only negative control in *in vitro* studies.

### Tissues and clinicopathological features of HCC

All of the human HCC tissues (n = 25) as well as the corresponding peritumoral (PT) non-tumor tissues (resected 1–2 cm from the malignant tumor) and the peripheral blood (n = 25) were obtained from HCC patients (Supplementary Table 1). The peripheral blood of healthy volunteers (n = 25) was obtained from the Immunohematology and Transfusion Medicine Service (Spedali Civili of Brescia, Italy). The study was approved by the ethical committee of Spedali Civili of Brescia on 2^nd^ October 2012 (NP1230) and informed consent was obtained from all the subjects enrolled in the study. All methods were performed in accordance with the relevant guidelines and regulations. Each biopsy specimen was confirmed to be either HCC or PT by pathological examination^21^. In this study, 30 HCC subjects underwent surgical resection at Spedali Civili, Surgical Clinic of Brescia (Italy). The subjects consisted of 24 men and 6 women ranging from 57 to 82 years of age. The subjects did not have any apparent distant metastases, and none had been previously treated for HCC. The patients were analyzed for the presence of the hepatitis B virus (HBV) or hepatitis C virus (HCV). Sixteen patients were positive for HCV, 4 were positive for HBV, and 10 were found to be negative for both HBV and HCV.

### LncRNAs and miRNAs profiling

The total RNA was isolated from cell cultures and from 200 µl of plasma using miRNeasy Mini Kit (Qiagen), according to the manufacturer’s instructions. The total RNA was isolated from tissue samples using TRIzol reagent (Invitrogen), according to the manufacturer’s instructions.

The RT^2^ lncRNA PCR Array Human lncFinder (Qiagen) and miScript miRNA PCR Array Human Liver miFinder (Qiagen) were employed to analyze the expression profile of different lncRNAs and miRNAs in HCC cell line (HA22T/VGH) treated or untreated with 15 μM sorafenib for 24 hours. In the first experiment we evaluated the expression changes of 84 candidate lncRNAs and 84 miRNAs by using RT^2^ lncRNA PCR Array Human lncRNA Finder and Human Liver miFinder miScript miRNA PCR Array (Qiagen) in HA22T/CGH cells treated and untreated with sorafenib according with manufacture’s instruction on 7500 Real‐Time PCR System. We tested 3 biological replicates and 3 technical replicates for both treated and untreated HA22T/VGH cells. Analysis of the RT‐qPCR data was performed using the free web-based PCR Array Data Analysis Software (available at https://dataanalysis.qiagen.com/pcr/; Qiagen). The relative expression levels of target lncRNAs/miRNAs were determined by the 2^−ΔΔCT^, method using 5 reference genes included in the array (for lncRNAs: RN7SK, RPLP0, ACTB, B2M, SNORA73A; for miRNAs: SNORD61 SNORD68 SNORD72 SNORD95 SNORD96A RNU6B/RNU6-2). Standard negative controls including “no template” and “no reverse transcriptase” reactions were also included in the arrays. The *P*-values were calculated by the software using a Student’s t-test for each gene in each treatment group compared to the control group.

### Quantitative real-time RT-PCR (qRT-PCR)

#### lncRNAs

cDNA was synthesized from 1 μg of total RNA using M-MLV Reverse Transcriptase (Invitrogen) according to the manufacturer’s instructions. The qPCR reaction was carried out using PrimeTime Gene Expression Master Mix (Integrated DNA technologies, IDT) and the appropriate PrimeTime qPCR Assay (20×) specific for the lncRNA targeted by the assay (either GAS5, HOXA-AS2 or HOTTIP). For circulating GAS5, 2.5 µl of total RNA from plasma was retrotranscribed and pre-amplified by using iScript Explore One-Step RT and PreAmp kit (BioRad) according to manufacturer’s instructions. The cDNA was diluted 1:10 and 1.5 µl were used for qPCR analysis together with appropriate PrimeTime qPCR Assay and 2× GoTaq Probe qPCR Master Mix (Promega). qPCR reactions were performed in triplicate. The expression levels of GAS5, HOXA-AS2 and HOTTIP were determined using the ΔΔCt method using RPLP0 as reference gene to normalize expression of target genes. HCC cases were stratified on the basis of decreasing GAS5 R values (R = RQ_HCC_/RQ_PT_). The GAS5 expression levels were considered to be “high” (R > 1.3), “unchanged” (0.7 < R < 1.3) and “low” (R < 0.7).

#### miRNAs

cDNA was synthesized from 50 ng of total RNA (or 3 µl purified RNA from plasma for circulating miR-126-3p) using the TaqMan microRNA Reverse Transcription Kit components (ThermoFisher) and the stem-loop primer for miR-126-3p (Applied Biosystems; Assay ID 002228) or RNU-48 (reference gene; Applied Biosystems; Assay ID 001006) and the spike-in cel-miR-39 according to the manufacturer’s instructions. qPCR reactions were performed in triplicate. The tissue expression levels of miR-126-3p were determined using the ΔΔCt method and RNU-48 as reference gene.

### Cell proliferation assay

The cells were seeded in a 96-well plate (5 replicates for each experimental condition) at a density of 2 × 10^3^ cells/well in complete medium and treated with increasing doses of sorafenib (0, 2.5, 5, 10, 15 μM). After 24, 48 and 72 hours, viability was assessed with the addiction of 15 μl/well of sterile CellTiter reagent (Promega). The plates were incubated at 37 °C for 2 hours in a humidified, 5% CO_2_ atmosphere and the absorbance at 490 nm was recorded using the microplate reader EnSight (PerkinElmer, Waltham, MA). The effects of miR-126-3p on cell viability in presence or absence of 15 μM sorafenib were evaluated by transfecting the cells with miR-126-3p mimics (Thermofisher, assay ID AM12841). Briefly, the cells were seeded in a 96-well plate (5 replicates for each experimental condition) at a density of 4 × 10^3^ cells/well in complete medium. After 24 h the cells were treated with 15 μM sorafenib or 0.1% DMSO. 24 h later the cells were transfected with 50/100 nM miR-126-3p mimics or 50/100 nM miR-NC (negative control #1 cat. n. 4464058) using lipofectamine reagent. After 24 and 48 hours, viability was assessed with the addiction of 15 μl/well of sterile CellTiter reagent. The plates were incubated at 37 °C for 2 hours in a humidified, 5% CO_2_ atmosphere and the absorbance at 490 nm was recorded using the microplate reader EnSight.

### Luciferase gene reporter assay

The presence of miR-126-3p binding site in the GAS5 sequence was predicted using the bioinformatics tool “IntaRNA” (http://rna.informatik.uni-freiburg.de/IntaRNA/Input.jsp)^22^.

The pmiRGLO Dual-Luciferase miRNA Target Expression Vector (Promega) was used to experimentally confirm the binding of miRNA by inserting the predicted miRNA target site of GAS5 at the 3′ of the firefly luciferase gene (*luc2*). The primers containing the putative binding site of miR-126-3p were custom synthesized (Integrated DNA Technologies) and are the following: Top S2: 5′-AAACTGATGGAGTCTCATGGCACAAGAAGATTAT-3′; Bottom S2: 5′-CTAGATAATCTTCTTGTGCCATGAGACTCCATCAGTTT-3′; Top AS2: 5′-AAATAATCTTCTTGTGCCATGAGACTCCATCAGT-3′; Bottom AS2: 5′-CTAGACTGATGGAGTCTCATGGCACAAGAAGATTATTT-3′.

The vector expressing the putative binding site was named pmiRGLO GAS2 S2, while the control vector with the corresponding sequence cloned in antisense orientation was named pmiRGLO GAS5 AS2. The empty vector was called pmiRGLO.

### Transient transfection and dual-luciferase reporter assay

The HA22T/VGH cells were seeded in a 24-well plate at a confluence of 60–80%. To evaluate the effect on miR-126-3p expression of GAS5 siRNA transfection upon sorafenib treatment, the cells were pre-treated for 24 h with 15 µM sorafenib and then transfected with 50 and 100 nM siRNA GAS5 and siRNA Negative Control (siRNA GAS5, cat #4390771 assay ID n272331; siRNA NC, cat #4390843, Life Technologies) using Lipofectamine 2000 transfection reagent (Life Technologies) according to the manufacturer’s instruction. 48 hours after transfection, the cells were lysed in TRIzol reagent (Life Technologies) and total RNA was extracted. For dual-luciferase reporter assay, the cells were transfected with 100 nM miR-126-3p mimic (Thermofisher) at 24 hours after seeding and after further 24 h, they were also transfected with 0.5 μg of luciferase reporter constructs using Lipofectamine 2000. Seventy-two hours after seeding, the cells were lysed by adding 100 μl of 1× passive lysis buffer (PLB) *per* well (Promega). The activity of Firefly (f-luc) and Renilla (r-luc) luciferases was evaluated by the Dual-Luciferase Reporter (DLR) Assay system (Promega) using the microplate reader EnSight (PerkinElmer).

### Statistical analysis

Statistical analysis was carried out using GraphPad Prism 7.0 software. Student’s t test was used to compare the expression of lncRNAs or miRNAs between the treated group and the untreated group with the sorafenib or to identify variations in cell proliferation. Diagnostic performance of circulating GAS5 and miR-126-3p to distinguish HCC patients from healthy subjects was evaluated using Receiver Operating Characteristic (ROC) curve analysis^23^. All experiments presented were performed at least three times. Data were considered statistically significant when *P*-value ≤ 0.05.

## Results

### The treatment of HCC cells with sorafenib led to lncRNAs and miRNAs dysregulation

To assess whether sorafenib may determine lncRNAs and miRNAs dysregulation, we examined the expression profiles of 84 disease- or pathway- focused lncRNAs and miRNAs in HA22T/VGH cells treated with 15 μM sorafenib or with 0.1% DMSO for 24 hours. Overall, 23 lncRNAs and 8 miRNAs resulted significantly dysregulated in sorafenib-treated HA22T/VGH cells compared to cells treated with DMSO. In particular, 3 lncRNAs were up-regulated while 20 were down-regulated (Supplementary Table 2) while regarding miRNAs, 3 were up-regulated and 5 down-modulated (Supplementary Table 3).

### GAS5, HOTTIP and HOXA-AS2 were dysregulated in HCC, renal and breast cancer cells following treatment with sorafenib

To verify the role of sorafenib in affecting the expression levels of ncRNAs, we selected and focused the attention on 3 lncRNAs and 1 miRNA for subsequent studies. GAS5 was chosen because it was up-regulated by the treatment while HOTTIP and HOXA-AS2 were both down-regulated. miR-126-3p was selected because it was the most down-regulated miRNA. We determined whether such ncRNAs were dysregulated in other HCC cells previously described to be sensitive to sorafenib (SKHep1C3, HuH6 and HepG2)^17^ and we expanded testing also to renal (ACHN, Caki-1 and CRBM 1990) and breast (MCF-7 and HCC 1937) cancer cells lines.

**Figure 1:**
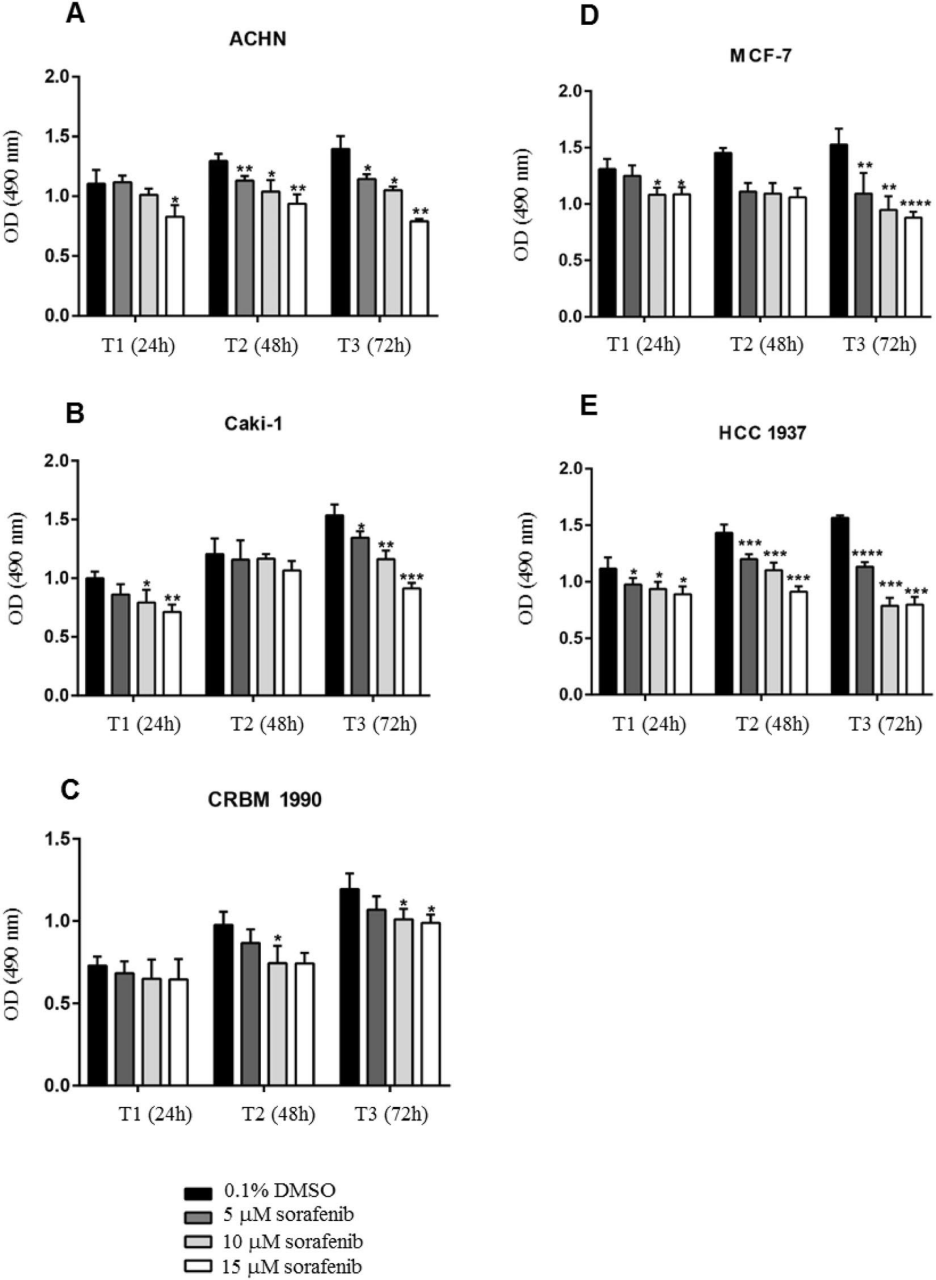
Sorafenib treatment inhibited the proliferation of renal and breast cancer cells. These bar charts show the effect of the sorafenib treatment on proliferation of ACHN (**A**), Caki-1 (**B**) and CRBM 1990 (**C**) renal cancer cells and of MCF-7 (**D**) and HCC 1937 (**E**) breast cancer cells. The difference between treated and untreated cells was evaluated for significance using unpaired t-test; *P-value < 0.05, **P-value < 0.01, ***P-value < 0.001, ****P-value < 0.0001. Bars represent the mean OD readings of samples measured at 490 nm and the lines represent the standard error of the mean (SEM).

Firstly, we evaluated whether the breast and renal cancer cells were sensitive to sorafenib by performing proliferation assay after treatment with increased concentrations of the drug (5, 10, 15 μM) for three days. Proliferation was assessed at 24 h, 48 h and 72 h post-drug exposure. Sorafenib significantly inhibited the proliferation of RCC (renal cancer cells) and breast cancer cells in a dose-dependent manner with proliferation decreasing with higher amount of drug used (Fig. 1A–E). However, CRBM 1990 cell line proliferation seemed to be affected to a lower degree at 72 h than the other cell line tested in which exposure to sorafenib had a more prominent effect on proliferation.

**Figure 2:**
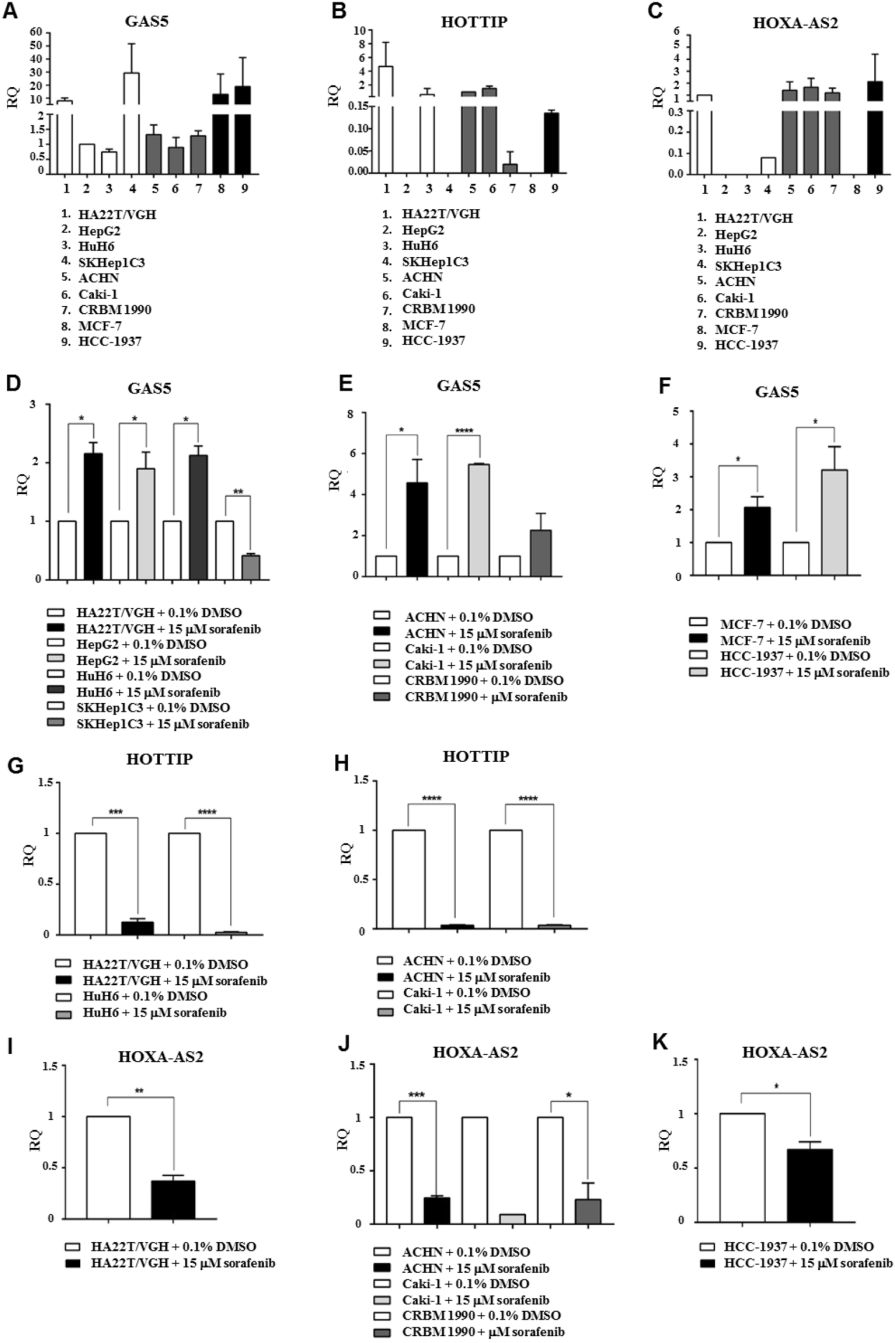
GAS5, HOTTIP and HOXA-AS2 were dysregulated in human cancer cells following treatment with sorafenib. The expression levels of GAS5 (**A**), HOTTIP (**B**) and HOXA-AS2 (**C**) in normal growth conditions were measured by qPCR in all of the HCC, renal and breast cancer cells used in this study. Expression of GAS5 (**D**–**F**), HOTTIP (**G**,**H**) and HOXA-AS2 (**I**–**K**) in cancer cells measured by qPCR when cells were exposed to 0.1% DMSO (open bar) or to 15 μM sorafenib (black or grey bars). GAS5 was upregulated in most HCC cell lines but SKHp1C3 (**D**) and in all but CRBM 1990 renal cells (**E**) and in all breast (**F**) cancer cells treated. HOTTIP resulted down-modulated in either HCC (**G**) or RCC (**H**) cells treated with 15 μM sorafenib. Similarly, HOXA-AS2 expression levels were down in hepatocellular (**I**), renal (**J**) and breast (**K**) cells exposed to sorafenib when compared with cells exposed to DMSO. Unpaired t-test was used to determine the presence of a statistically significant difference between cells exposed to sorafenib and cells exposed to DMSO only: *P-value < 0.05, **P-value < 0.01, ***P-value < 0.001; ****P-value < 0.0001. The bars represent the mean expression values from 3 experiments and the lines represent the standard error of the mean (SEM).

Subsequently, we assessed the amount of the selected lncRNAs in the cancer cells used in this study in normal growth condition. GAS5 expression was detected in all the cells analyzed but with some cell lines expressing higher levels than others (Fig. 2A). HOTTIP was expressed at very low levels in HuH6, CRBM 1990 and HCC 1937 cells, while it was not expressed in HepG2, SKHep1C3 and MCF-7 cells (Fig. 2B). HOXA-AS2 was expressed at low levels in all the cell lines but HepG2, HUH6 and MCF7 did not show any expression (Fig. 2C).

In order to assess the trend of GAS5 expression after sorafenib treatment, we measured the GAS5 levels by qPCR in the aforementioned cell lines and treated with 15 μM sorafenib for 24 hours. As control, we exposed the cells to 0.1% DMSO for the same amount of time. GAS5 was significantly up-regulated in 7 cell lines out of 9 (HA22T/VGH, HepG2, HUH6, ACHN, Caki-1, MCF-7 and HCC 1937) treated with sorafenib compared to cells treated with DMSO only (Fig. 2D–F). In CRBM 1990 cells, GAS5 was up-regulated when treated with the drug but the difference was not significant. On the contrary, GAS5 was significantly down-modulated in SKHep1C3 cells when exposed to sorafenib. The results obtained in HA22T/VGH cells were in line with the expression profile determined by lncRNA PCR array proving that sorafenib may mediate GAS5 up-regulation in other HCC cells as well as in renal and breast cancer cells. To verify a dose-dependent response between sorafenib treatment and GAS5 upregulation, HA22T/VGH cells were treated with 5, 10 and 15 µM sorafenib for 24 h. GAS5 resulted upregulated 2, 2.3 (*P*-value < 0.05) and 2.4 (*P*-value < 0.01) fold, respectively when compared to 0.1% DMSO treated cells (Supplementary Fig. 1A).

HOTTIP was significantly down-regulated in the 4 cell lines tested (HA22T/VGH, HUH6, ACHN and Caki-1) when they were treated with sorafenib compared to cells treated with 0.1% DMSO (Fig. 2G,H). These data were in agreement with the HOTTIP expression profile obtained by lncRNA PCR array in HA22T/VGH cells and indicated that sorafenib may also mediate HOTTIP down-regulation in another HCC cell line and in two renal cancer cell lines. In addition, HOTTIP showed a trend of down-regulation in the HCC 1937 and CRBM 1990 cell lines, but the expression levels were at the limit of detection (data not shown). As shown in Fig. 2B, the other cell lines (HepG2, SKHep1C3 and MCF-7) did not express HOTTIP.

HOXA-AS2 was significantly down-regulated in 4 cell lines out of 5 (HA22T/VGH, ACHN, CRBM 1990 and HCC 1937) treated with sorafenib compared to DMSO-treated cells (Fig. 2I–K). The data were in accordance with the HOXA-AS2 expression profile obtained by lncRNA PCR array in HA22T/VGH cells and indicated that sorafenib may mediate HOXA-AS2 down-modulation also in renal and breast cancer cells. In Caki-1 cells, HOXA-AS2 seemed to be down-regulated although the difference was not statistically significant. As shown in Fig. 2C, the other cell lines did not express or expressed very low levels of HOXA-AS2 and could not be used for these experiments.

### miR-126-3p was differentially expressed in human cancer cells and it was down-regulated in HCC, renal and breast cancer cells following sorafenib treatment

It was previously established by qPCR array that miR-126-3p was affected by sorafenib and in particular it was down-regulated in HA22T/VGH cells treated with the drug. Therefore we set out to find if sorafenib affected this miR in other cell lines in a similar way.

**Figure 3:**
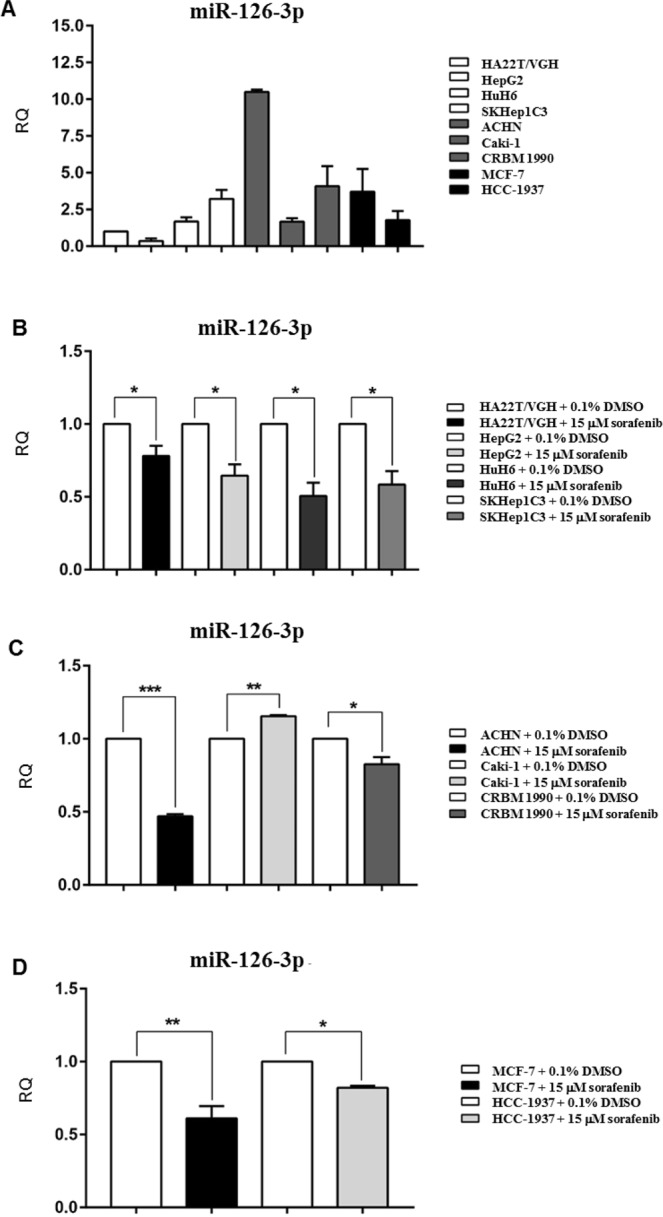
miR-126-3p was differentially expressed in HCC, renal and breast cancer cells and it was mostly down-regulated following the treatment with sorafenib. The expression levels of miR-126-3p in normal growth conditions were determined by qPCR in the cell lines used in this study (**A**). Expression of miR-126-3p in cancer cells was measured by qPCR when cells were exposed to 0.1% DMSO (open bar) or to 15 μM sorafenib (black or grey bars). miR126-3p expression levels were down-regulated in all hepatocellular (**B**) and breast (**D**) cells and in most of renal (**C**) cells exposed to sorafenib when compared with cells exposed to DMSO. The only exception was Caki-1 where it was up-regulated (**C**). Unpaired t-test was used to determine the presence of a statistically significant difference between cells exposed to sorafenib and cells exposed to DMSO only: *P-value < 0.05, **P-value < 0.01, ***P-value < 0.001; ****P-value < 0.0001. The bars represent the mean expression values from 3 experiments and the lines represent the standard error of the mean (SEM).

Firstly, we measured its expression in normal growth condition with the panel of cells used in this work. As shown in Fig. 3A, miR-126-3p was expressed at various levels in the cells analyzed. Subsequently, we quantified the expression of miR-126-3p after treatment with 15 μM sorafenib for 24 hours using the same cell lines (Fig. 3B–D). This miRNA was significantly down-regulated in 8 cell lines out of 9 treated with sorafenib compared to the cells exposed to DMSO. On the contrary, it was significantly up-regulated in Caki-1 cells. Taken together, these data indicate that sorafenib was able to inhibit miR-126-3p expression in several cell lines from different cancer types, paralleling the results obtained in the HA22T/VGH cells. To verify a dose-dependent response between sorafenib treatment and miR-126-3p down-modulation, HA22T/VGH cells were treated with 5, 10 or 15 µM sorafenib for 24 h. miR-126-3p resulted 16%, 25% (*P*-value < 0.05) and 24% (*P*-value < 0.01) down-regulated compared to 0.1% DMSO treated cells (Supplementary Fig. 1B). Furthermore, the GAS5 knockdown induced by 50 nM or 100 nM specific siRNA transfection determined a significant increase of miR-126-3p expression in the same cell line upon sorafenib treatment (*P*-value < 0.05 and *P*-value < 0.01 *versus* 50 nM or 100 nM siRNA NC, respectively; Supplementary Fig. 1C,D). These data suggested that the targeting of GAS5 resulted in the up-regulation of miR-126-3p levels upon sorafenib treatment. This finding could suggest that GAS5 may be involved in the regulation of miR-126-3p expression in HA22T/VGH cells treated with sorafenib.

### GAS5, HOTTIP, HOXA-AS2, miR-126-3p were dysregulated in sorafenib resistant cells

**Figure 4:**
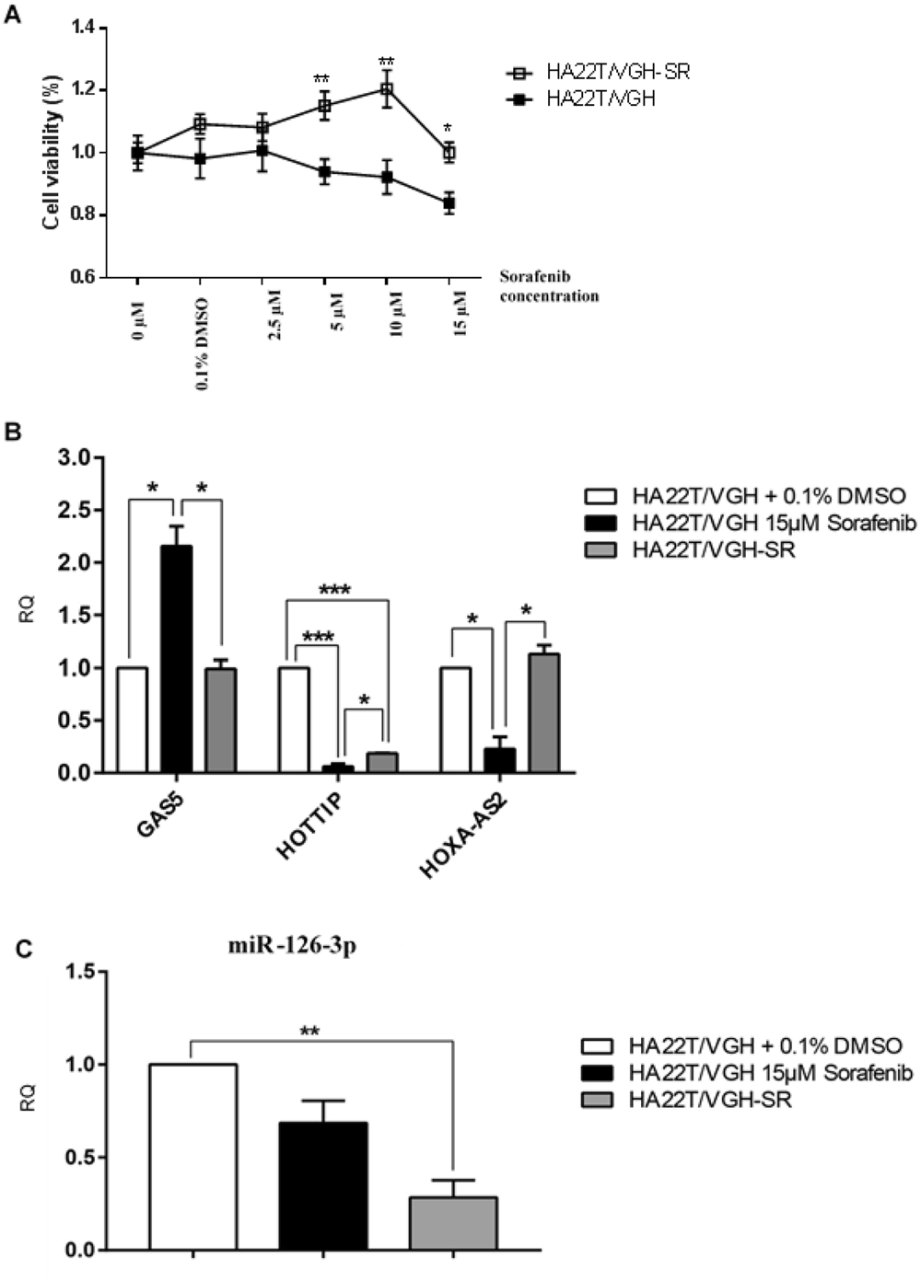
GAS5, HOTTIP, HOXA-AS2 and miR-126-3p were dysregulated in sorafenib resistant HCC cells. (**A**) This graph displays the effects of sorafenib treatment on the proliferation of HA22T/VGH resistant cells (HA22T/VGH-SR) compared with the parental sensitive ones. (**B**) Expression of GAS5, HOTTIP, HOXA-AS2 or (**C**) miR-126-3p in HA22T/VGH sensitive cells or HA22T/VGH-SR resistant cells exposed to 15 μM sorafenib and in HA22T/VGH cells exposed to 0.1% DMSO. Unpaired t-test was used to determine the presence of a statistically significant difference between either sensitive or resistant cells exposed to sorafenib and sensitive cells exposed to DMSO only: *P-value < 0.05, **P-value < 0.01, ***P-value p < 0.001; ****P-value < 0.0001. The bars represent the mean expression values from 3 experiments and the lines represent the standard error of the mean (SEM).

In order to verify whether the selected ncRNAs may be dysregulated after the acquisition of resistance to sorafenib, we assessed their expression levels and the cell viability in a sorafenib-resistant cell line derived from HA22T/VGH cells and labelled HA22T/VGH-SR after exposure to the drug. As shown in Fig. 4A, sorafenib clearly elicited distinct effects on cell proliferation with resistant cells maintaining or increasing their viability with incremental concentrations of sorafenib up to 10 μM while sensitive cells started to drop their viability after the concentration of 2.5 μM. This demonstrated that HA22T/VGH-SR cells were resistant to sorafenib treatment until 10 μM dose concentration and showed a 31% proliferation increase (*P*-value ≤ 0.001) in HA22T/VGH-SR cells respect to the sensitive ones.

We compared the expression levels of ncRNAs in resistant cells to the sensitive ones when they were both treated with 15 μM sorafenib for 24 hours and normalized against the expression levels of HA22T/VGH exposed to 0.1% DMSO. GAS5 expression levels were significantly down-regulated in HA22T/VGH-SR compared to HA22T/VGH cells treated with sorafenib (Fig. 4B), while HOXA-AS2 expression was significantly up-regulated. HOTTIP expression levels were downregulated in both sensitive and resistant cells compared to HA22T/VGH cells treated with DMSO although resistant cells showed a slightly higher and significant levels of expression than the sensitive cells when treated with sorafenib (Fig. 4B). These results may suggest that the acquisition of resistance to sorafenib in HA22T/VGH-SR cells may be connected to the restoration of GAS5 and HOXA-AS2 expression to the levels found in the sensitive and untreated cells.

Results with miR-126-3p showed that it was downregulated in HA22T/VGH-SR cells compared to either the cells exposed to DMSO or to HA22T/VGH exposed to sorafenib (Fig. 4C). This may suggest that acquisition of resistance to sorafenib may lead to a further downregulation of this miRNA.

### miR-126-3p directly interacted with GAS5 in HA22T/VGH cells

**Figure 5:**
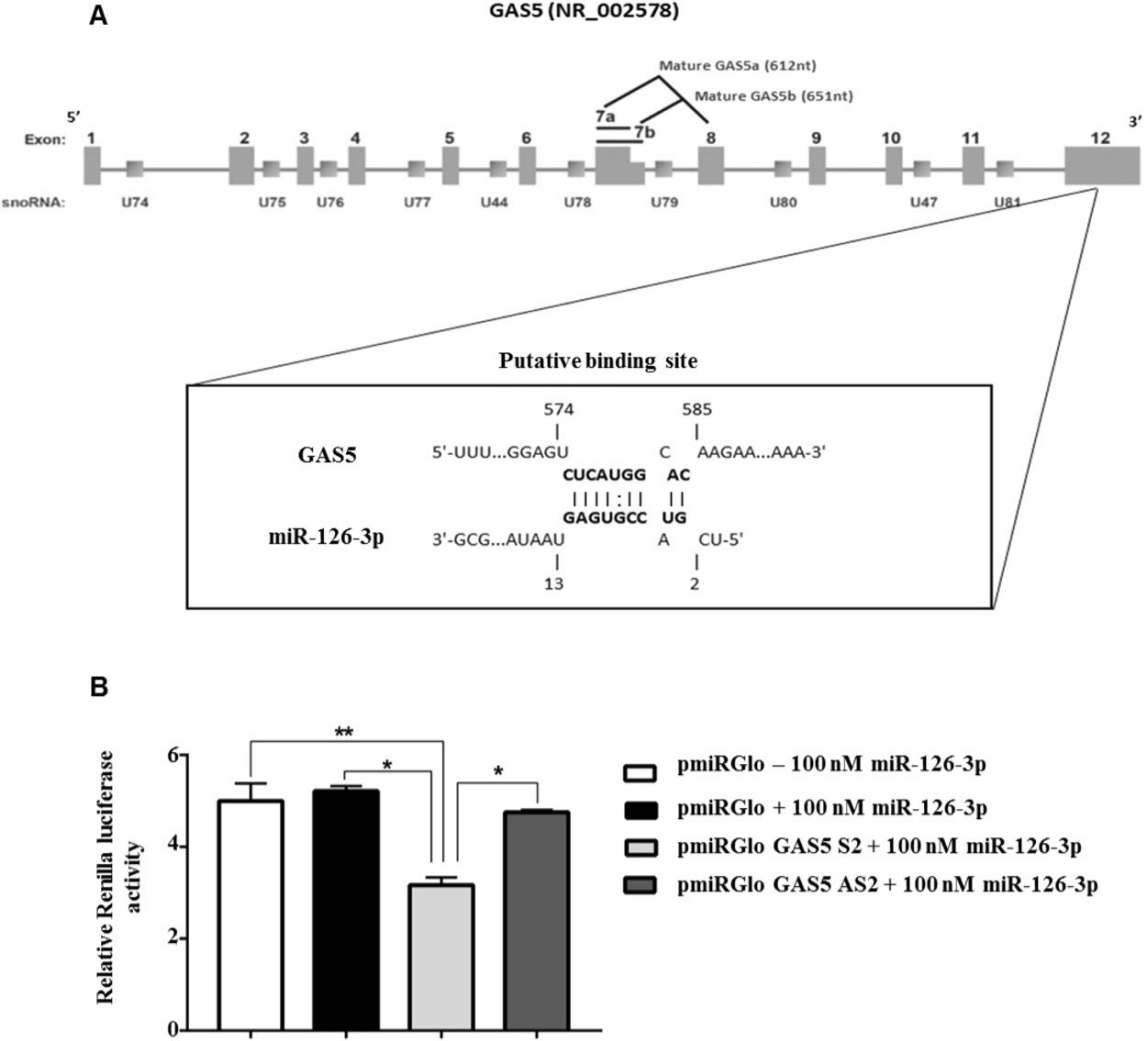
miR-126-3p may directly interact with a putative binding site found in GAS5 in HA22T/VGH cells. (**A**) Bioinformatics analysis showed that GAS5 may harbour a putative binding site for miR-126-3p; the location and the complementarity between miR-126-3p and the putative GAS5 3′-UTR target site are shown in the enlarged inset. (**B**) Luciferase activity measured in HA22T/VGH cells following ectopic expression of miR-126-3p and co-transfection with pmiRGLO, pmiRGLO GAS5 S2 or pmiRGLO GAS5 AS2. Unpaired t-test was used to determine the presence of a statistically significant difference between the cells transfected with different constructs: *P-value < 0.05, **P-value < 0.01, ***P-value p < 0.001; ****P-value < 0.0001. The bars represent the mean luciferase activity values from 3 experiments and the lines represent the standard error of the mean (SEM).

Since 7 out of 9 cell lines treated with sorafenib (Figs 2D–F; 3B–D) displayed opposite expression levels between the up-regulated GAS5 and the down-regulated miR-126-3p and the knockdown of GAS5 promoted the increase of miR-126-3p expression in sorafenib treated HA22T/VGH cells (Supplementary Fig. 1C,D), we explored whether GAS5 may act as a ceRNA for miR-126-3p. To do that, we performed bioinformatics analysis using the algorithm IntaRNA (http://rna.informatik.uni-freiburg.de/IntaRNA/Input.jsp) that predicted a putative miR-126-3p binding site (BS) within the exon 12 of GAS5 (nt 575-584; ref. n. NR_002578; Fig. 5A).

To verify a potential direct molecular interaction, a 34-bp long sequence of GAS5 containing the putative miR-126-3p BS was cloned into the pmiRGLO vector (pmiRGLO GAS5 S2). A control construct was obtained by cloning the same fragment in an antisense orientation (pmiRGLO GAS5 AS2). HA22T/VGH cells were then transfected with 100 nM miR-126-3p mimic and with the obtained constructs (pmiRGLO, pmiRGLO GAS5 S2 and pmiRGLO GAS5 AS2). The amounts of luciferase activity detected in HA22T/VGH cells with empty vector with or without miR-126-3p mimic and with antisense construct with miR-126-3p mimic were very similar. However, the pmiRGLO GAS5 S2 construct in presence of miR-126-3p mimic showed a 39% decrease in the luciferase activity compared to the control empty vector without mimic compound (Fig. 5B). The luciferase activity measured here was significantly lower (*P*-value ≤ 0.01) compared to the one measured for any other sample. The data showed that the putative BS of the GAS5 was recognized by miR-126-3p and therefore it is possible that GAS5 may act as a sponge for miR-126-3p.

### Effects of miR-126-3p ectopic expression on cell viability in HA22T/VGH treated or not treated with sorafenib

**Figure 6:**
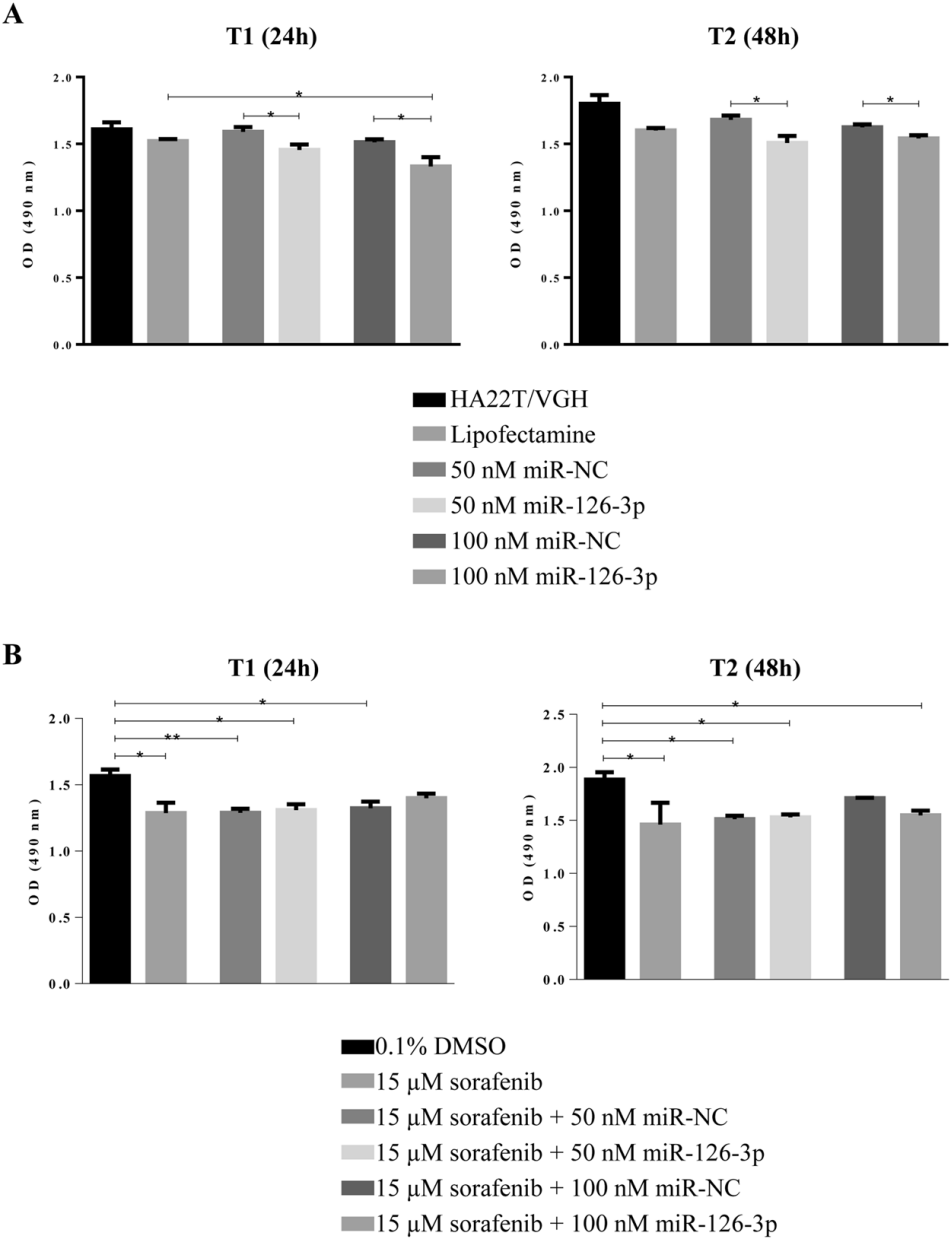
Effects of miR-126-3p ectopic expression on HA22T/VGH cells growth untreated/treated with sorafenib. (**A**) MTT assay was performed in HA22T/VGH cells transfected with 50 nM or 100 nM of miR-NC or miR-126-3p. The ectopic expression of miR-126-3p significantly decreased cell growth after 24 hours and 48 hours from transfection compared to corresponding controls. *P-value < 0.05 *versus* Lipofectamine or miR-NC in t-Test analysis for unpaired comparison. (**B**) The HA22T/VGH cells were treated with 15 µM sorafenib or vehicle (0.1% DMSO) and transfected with miR-NC or miR-126-3p (50 nM or100 nM). MTT assay showed that sorafenib significantly reduced cell viability at 24 hours and 48 hours after miRs transfection. No additive effects were observed using the ectopic expression of miR-126-3p. One-way ANOVA followed by Bonferroni test as pair-wise post-hoc test was used to test the significant difference in expression btween the different groups: **P-value < 0.01, *P-value < 0.05. The histograms represent the mean expression values from 5 replicates for each conditions and the lines represent the standard error of the mean (SEM). These graphs are representative of one of two experiments.

In order to verify whether miR-126-3p over-expression affected HA22T/VGH cell growth, we transfected the cells with miR-126-3p mimics. miRNA-126-3p ectopic expression significantly inhibited the HA22T/VGH cell viability at 50 and 100 nM concentration compared to the corresponding doses of miR-NC (Fig. 6A) at 24 h and 48 h after transfection.

Since sorafenib mediated the down-modulation of miR-126-3p in tumor cells, we assessed the effects on cell growth of miR-126-3p ectopic expression following sorafenib treatment of HA22T/VGH cells. As expected, a significantly decreased of proliferation ability was observed for cells treated with 15 µM sorafenib. However, the concomitant overexpression of miR-126-3p did not affect the proliferation inhibition any further (Fig. 6B). These data would exclude an additive effect using miR-126-3p and sorafenib treatment on HA22T/VGH cell proliferation.

### Expression of miR-126-3p in tissues and plasma of HCC patients and plasmatic detection of GAS5

**Figure 7:**
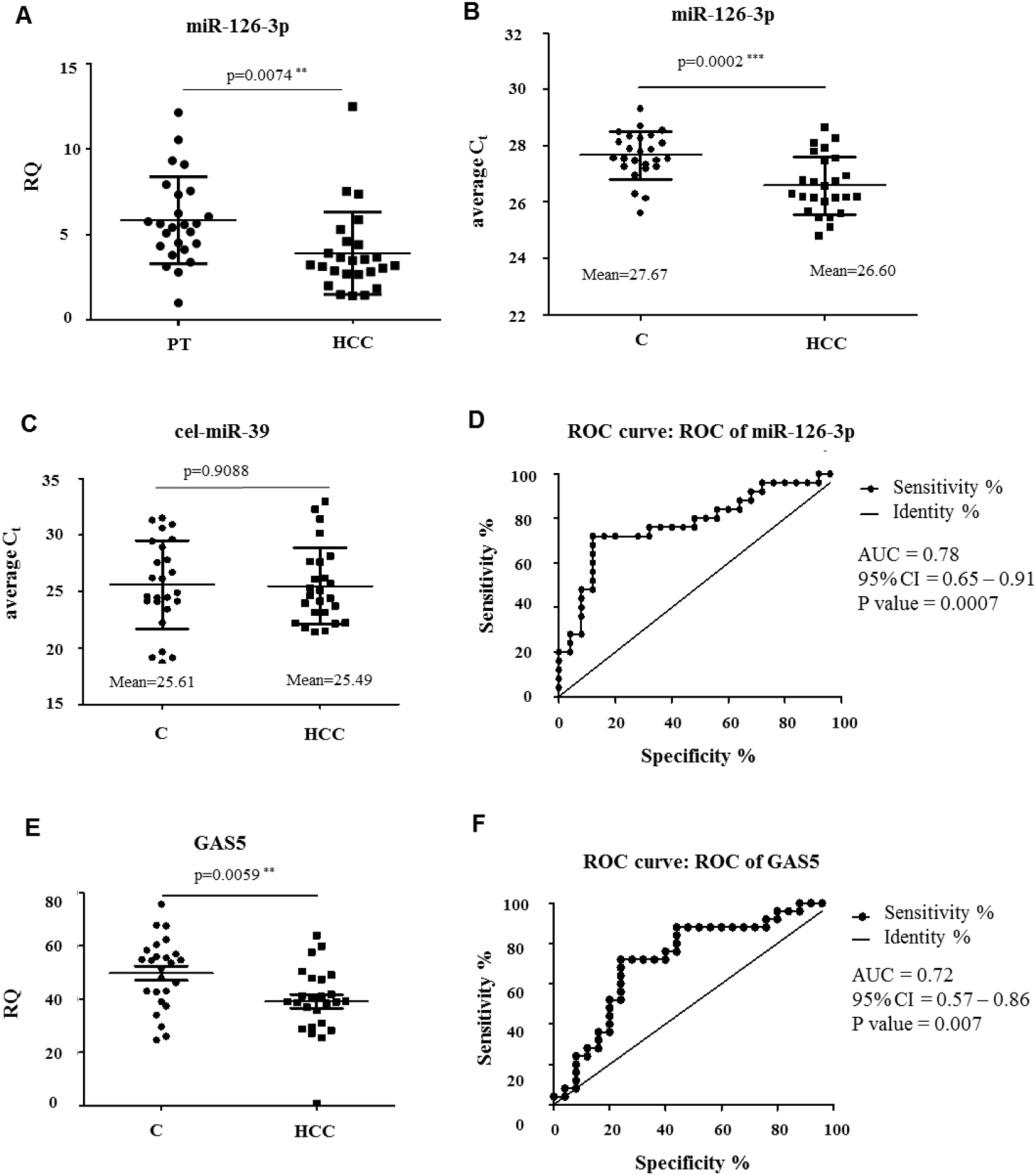
miR-126-3p levels in plasma and in tissues and circulating GAS5 in plasma from HCC patients and healthy subjects. (**A**) miR-126-3p levels were significantly down-modulated in HCC compared to PT (n = 25). Dotplots of the average C_t_ of the circulating miR-126-3p (**B**) and the spike-in cel-miR-39 (**C**) in healthy subjects and in HCC patients; HCC patients showed higher levels of miR-126-3p in plasma than healthy controls. (**D**) ROC curve analysis of plasma miR-126-3p. AUC = area under the ROC curve; CI = confidence interval. (**E**) Dotplot of the circulating GAS5 for healthy subjects or HCC patients; GAS5 levels in HCC patients were significantly lower compared to control subjects. (**F**) ROC curve analysis of plasma GAS5. AUC = area under the ROC curve; CI = confidence interval. Two-tailed, paired t-test was used to determine the presence of a statistically significant difference between ncRNAs expression in HCC patients against healthy controls or between HCC tumour and peritumoral areas: *P-value < 0.05, **P-value < 0.01, ***P-value p < 0.001; ****P-value < 0.0001.

Since miR-126-3p was significantly down-modulated after sorafenib treatment in almost all the cells included in the present study, we assessed its expression levels in HCC tissues and plasma from HCC patients to better understand the variation in its expression. miR-126-3p was significantly down-regulated in HCC tissues compared to their matched peritumoral (PT) tissues using qPCR analysis (RQ_HCC_ average = 3.91 ± 0.48 *vs*. RQ_PT_ average = 5.84 ± 0.51; *P*-value = 0.0074; Fig. 7A). Regarding the expression of GAS5 in the same cohort, we did not find any significant correlation with miR-126-3p expression. However, we observed a high percentage (64%) of cases with simultaneously high GAS5 expression and low miR-126-3p expression (Supplementary Fig. 2). The levels of circulating miR-126-3p were significantly higher in HCC patients compared to controls (Fig. 7B). The plotting was made on the average raw cycle thresholds (C_t_) since there were no established endogenous miRNAs acting as normalizers for plasma miRs. The average Ct values of the spike-in cel-miR-39 were the same in the two groups demonstrating good and constant performance of RT and qPCR reactions (Fig. 7C). The ROC curve analysis evidenced the possibility to use the plasmatic miR-126-3p levels to distinguish HCC patients from control subjects (AUC = 0.78; *P*-value = 0.0007; Fig. 7D).

In order to assess whether GAS5 was released into the bloodstream, we determined its level in the plasma by qPCR. The expression levels of GAS5 were significantly lower in HCC patients compared to controls (Fig. 7E). The ROC curve analysis evidenced the possibility to use GAS5 to distinguish HCC patients from control subjects (AUC = 0.72; *P*-value = 0.007; Fig. 7F).

## Discussion

The main aim of this work was to investigate whether the oral multikinase inhibitor sorafenib could modulate the expression of given ncRNAs in HCC cells. The qPCR array study revealed that a total of 23 lncRNAs (3 up-regulated and 20 down-regulated) and 8 miRNAs (5 down-modulated and 3 upregulated) were significantly dysregulated in sorafenib-treated HCC cells (HA22T/VGH) when they were compared to untreated cells. These findings pointed to the evidence that sorafenib affected mainly the expression levels of the lncRNAs rather than miRNAs included in the array. To our knowledge, this is the first study designed to examine the expression changes of a wide number of lncRNAs after sorafenib treatment of cultured cells. We focused on the expression of GAS5 and HOXA-AS2 since they were the most upregulated and down-modulated genes, respectively and on the expression of HOTTIP because its down-modulation was at intermediate level. We also chose miR-126-3p because it was the most down-modulated miR.

The expression levels of GAS5 were found to be increased in almost all the cancer cells treated with sorafenib compared to cells exposed to DMSO (except SKHep1C3) proving that sorafenib may up-regulate GAS5 not only in different HCC cell lines but also in renal and breast cancer cells. It is well known that GAS5 acts as tumor-suppressor in several malignancies and it was reported to be significantly lower in clinical renal cell carcinoma (RCC) specimens^24^, breast cancer tissues^25^ and HCC tissues^26^ when compared to the adjacent normal tissues. Instead, very few data are present in the literature about circulating GAS5 in human malignancies. For example, high GAS5 serum levels were associated with best prognosis in patients with glioblastoma^27^ while GAS5 levels were found to be decreased in serum^27^ and in plasma^28^ of colorectal cancer (CRC) patients compared to control subjects. The plasma levels of GAS5 of HCC patients and control individuals were compared for the first time in the present study. Significant lower levels of GAS5 were found in the plasma of HCC patients in comparison with control subjects and the ROC analysis evidenced the potential ability of circulating GAS5 levels to discriminate HCC patients from control subjects.

Regarding HOTTIP, 4 tumor cell lines (HA22T/VGH, HuH6, ACHN and Caki-1) among the ones tested expressed appreciable levels of this lncRNA and in which HOTTIP was significantly down-regulated following treatment with sorafenib. It is known that HOTTIP can exert oncogenic effects in solid tumors^29^ and has been found significantly upregulated in various types of human cancer, including hepatocellular carcinoma, pancreatic, gastric and colorectal cancer. It has also being associated with metastasis, progression and prognosis in several neoplastic diseases^30–32^. Similar considerations can be extended to the lncRNA HOXA-AS2. In fact, the cancer cell lines that exhibited the highest expression levels of this gene in normal growth condition also displayed its down-modulation after sorafenib treatment (HA22T/VGH, ACHN, Caki-1, CRBM 1990 and HCC1937). HOXA-AS2 was previously found to be significantly up-regulated in breast cancer tissues^33^ and HCC tissues compared with normal tissues^34^ and to promote tumorigenesis of HCC^34^.

The results obtained in the present study indicate that sorafenib mediated the up-regulation of GAS5 and the down-regulation of HOTTIP and HOXA-AS2 in most HCC, renal and breast cancer cells and probably their expression modulation may lead to a less aggressive cancer phenotype of the cells. Subsequent studies will aim to address this issue.

It has been established that some HCC patients are either initially resistant to sorafenib or become resistant to it during its use^35^ reducing the efficacy of the treatment. Therefore, efforts have been directed to identify novel predictive molecular biomarkers for primary and/or acquired resistance to sorafenib and to discover novel strategies to improve its effectiveness also by understanding how lncRNAs expression are affected by it. The lncRNAs are known to be involved in chemotherapy and drug resistance or response. Recently, Xue *et al*. have found that HOTAIR is highly upregulated in the tumors of tamoxifen-resistant breast cancer patients compared to their primary counterparts^36^. Liu *et al*. identified that GAS5 was downregulated in sorafenib non-responsive RCCs and that GAS5 overexpression conferred sensitivity to sorafenib in nonresponsive RCC cells^18^. Again, Jean *et al*. found that knockdown of lncRNA TUC338 in HepG2 rendered these cells sensitive to the treatment with sorafenib^37^. In this context, we have found that the sorafenib resistant cells HA22T/VGH-SR (obtained after months of continuous treatment with increasing concentration of sorafenib) expressed levels of GAS5 and HOXA-AS2 comparable to those of sensitive and untreated cells indicating that the cells gradually restored the expression levels of these genes during resistance development. Thus, the quantification of the expression levels of these 2 lncRNAs during the treatment may indicate the onset of drug resistance in HCC cells. It is conceivable that GAS5 and HOXA-AS2 dysregulation occurred not only following sorafenib response, but also after the acquisition of sorafenib-resistance in HA22T/VGH cells.

In the present study, we also reported that sorafenib influenced miR-126-3p expression since it resulted down-modulated following sorafenib treatment in all the cells except Caki-1 cells. miR-126 have been shown to have a significant impact on many human cancer types acting as a tumor suppressor in several tumors. Lower expression of miR-126 in HCC was significantly associated with tumor recurrence and poor survival of patients^38^. miR-126-3p upregulation have been associated with decreased cell proliferation, apoptosis induction, and inhibition of tumor angiogenesis in HCC^39^. In this context, miR-126-3p ectopic expression decreased the growth capacity of HA22T/VGH cells, but the miR-126-3p over-expression in sorafenib-treated HA22T/VGH did not affect further the proliferation inhibition promoted by sorafenib. It is challenging to reconcile the observation that miR-126-3p generally has tumor suppression functions with the observation that miR-126-3p expression levels further decreased in sorafenib sensitive and also in drug-resistant cells after sorafenib treatment. Consequently to better elucidate its role, we measured miR-126-3p levels in HCC tissues from biopsies and we quantified its plasmatic circulating levels. miR-126-3p resulted significantly down-regulated in HCC tissues respect to their peritumoral counterparts (PT), in agreement with other previously reported data^39,40^. The levels of plasmatic circulating miR-126-3p were higher in HCC patients respect to healthy subjects and this has the potential to be used to discriminate HCC patients from healthy people as found by receiver operating characteristic (ROC) curve analysis. To the best of our knowledge, only three previous publications reported data about circulating miR-126 levels. Gosh *et al*. showed increased miR-126-3p expression in plasma of HBV-positive/HCC patients compared to HBV-positive/non-HCC patients^41^ in accordance with our results. In other two published reports, the miR-126 circulating levels were measured in serum. Khairy *et al*. found a significant decrease of levels of miR-126 in the serum of HCC patients compared to non-HCC group^42^. Ali *et al*. demonstrated that the median serum level of miR-126-3p was significantly reduced in HCC patients compared to healthy controls^43^. In summary, miR-126-3p has been shown to be upregulated in plasma but down-modulated in serum of HCC patients. This should not be surprising, since the differences in miRs levels between plasma and serum have been commonly described^44^. The question that remain to answer is why miR-126-3p decreased following sorafenib treatment in our cancer *in vitro* models. At the present, we may speculate that miR-126-3p marginally contributes to the less aggressive phenotype of cancer cells observed after drug treatment (*i.e*. in terms of proliferation inhibition). However, the trend of miR-126-3p down-modulation obtained in all the treated cells would suggest that sorafenib may determine the expression variation of this miRNA very likely indirectly. We think that miR-126-3p levels should be further investigated in patients treated or not with sorafenib to verify this hypothesis.

Finally, we found by bioinformatics analysis that GAS5 had a putative binding site for miR-126-3p and demonstrated by luciferase reporter assay a direct interaction between GAS5 and miR-126-3p. It has been previously shown that GAS5 may function as miRNA sponge or competing endogenous RNA (ceRNA) for some miRs included miR-21^45^ and miR-222^46^ and therefore we hypothesized that GAS5 may act as a sponge for miR-126-3p in HCC cells. In support to this hypothesis, we found that the silencing of GAS5 resulted in miR-126-3p up-regulation upon sorafenib treatment in the rescue experiment. In addition, 64% of the HCC patients with high GAS5 expression had also miR-126-3p down-modulated.

Circulating ncRNAs have been described in different human body fluids, including serum/plasma and urine and are promising biomarkers for cancer risk assessment, diagnosis, prognosis, and to monitor the treatment response^47–50^. In this context, our results on circulating GAS5 and miR-126-3p would indicate their potential to be used to discriminate HCC patients from healthy subjects. These results may be considered an initial step in the identification of novel non-invasive circulating biomarkers of disease. Future studies will be addressed to verify whether GAS5 and miR-126-3p may be potential early/predictive circulating biomarkers in at high-risk patients (i.e. patients with cirrhosis and/or HBV, HCV chronic hepatitis).

In conclusion, elucidation of the downstream effects of sorafenib treatment may be useful to better understand novel aspects of its mechanism of action that has not been yet completely elucidated and therefore to augment the basic knowledge of intracellular events occurring in cancer cells after sorafenib treatment.

## Supplementary information

Supplementary Info
