# Supplementary material for: Differential expression profiling of long non-coding RNA GAS5 and miR-126-3p in human cancer cells in response to sorafenib
Source: Sci Rep. 2019 Jun 24;9:9118. doi: 10.1038/s41598-019-45604-2 (PMC6591391; doi:10.1038/s41598-019-45604-2)
Supplement: Supplementary file 1 — Supplementary Info [file 41598_2019_45604_MOESM1_ESM.docx]

**Supplementary Figure 1. Expression level of GAS5 and miR-126-3p in HA22T/VGH cells following sorafenib treatment and siRNA GAS5 transfection.**

Differential expression profiling of long non-coding RNA GAS5 and miR-126-3p in human cancer cells in response to sorafenib.

*Teresa Faranda, Ilaria Grossi, Michele Manganelli, Eleonora Marchina, Gianluca Baiocchi, Nazario Portolani, Marialuisa Crosatti, Giuseppina De Petro, Alessandro Salvi.*

A

B

C

D

GAS5

GAS5

miR-126-3p

miR-126-3p

**Supplementary Figure 2. Waterfall plot of GAS5 and miR-126-3p expression levels.**

Differential expression profiling of long non-coding RNA GAS5 and miR-126-3p in human cancer cells in response to sorafenib.

*Teresa Faranda, Ilaria Grossi, Michele Manganelli, Eleonora Marchina, Gianluca Baiocchi, Nazario Portolani, Marialuisa Crosatti, Giuseppina De Petro, Alessandro Salvi.*


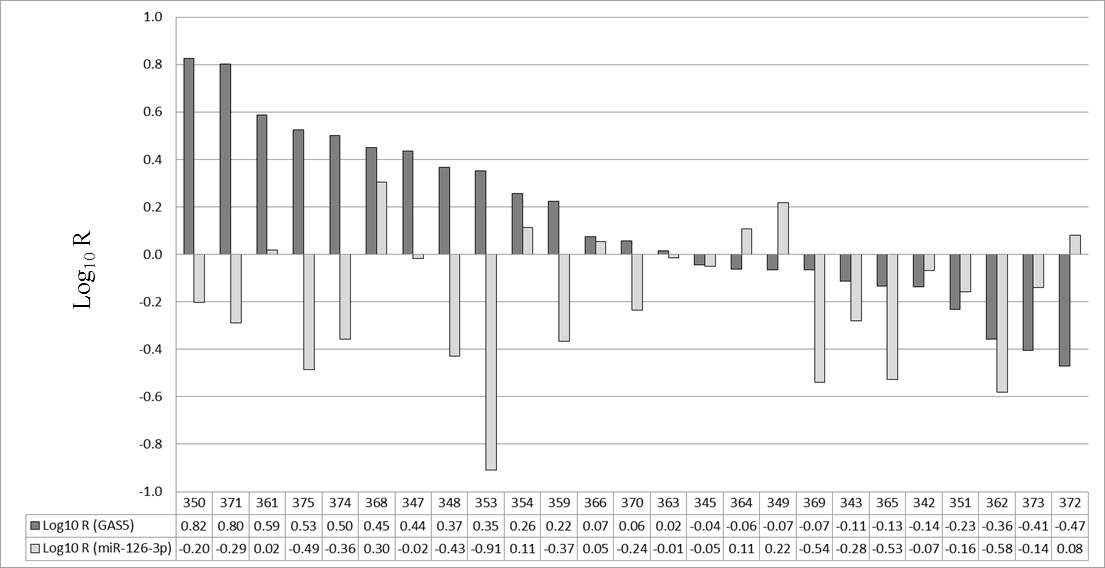


**SUPPLEMENTARY FIGURE LEGENDS**

Differential expression profiling of long non-coding RNA GAS5 and miR-126-3p in human cancer cells in response to sorafenib.

*Teresa Faranda, Ilaria Grossi, Michele Manganelli, Eleonora Marchina, Gianluca Baiocchi, Nazario Portolani, Marialuisa Crosatti, Giuseppina De Petro, Alessandro Salvi.*

**Supplementary Figure 1. Expression level of GAS5 and miR-126-3p in HA22T/VGH cells following sorafenib treatment and siRNA GAS5 transfection**. (A) GAS5 expression levels were significantly up-regulated following 10 µM or 15 µM sorafenib treatment. (B) miR-126-3p expression levels were significantly down-modulated following 10 µM or 15 µM sorafenib treatment. (C) Knockdown efficiency of GAS5 siRNA transfection in HA22T/VGH cells treated with 15 µM sorafenib. (D) miR-126-3p expression levels were significantly up-regulated after 48 hours from 50 nM or 100 nM GAS5 siRNA transfection in HA22T/VGH cells treated with 15 µM sorafenib. Unpaired two-tailed t-test was used to determine the presence of a statistically significant difference in expression between each group: **P*-value < 0.05, ***P*-value < 0.01, ****P*-value < 0.001. Histograms are the means and the bars are ± SEM.

**Supplementary Figure 2. Waterfall plot of GAS5 and miR-126-3p expression levels.** Differential expression of the lncRNA GAS5 and the miR-126-3p in 25 HCC human tissue biopsies. Histograms represent R-values. The R-value is the HCC *vs* PT ratio of the relative quantification (RQ) of GAS5 and miR-126-3p expression levels respectively in each case. Of note, 11 cases showing an over-expression of GAS5 displayed a down-modulation of miR-126-3p. The same trend was not observed in the remaining cases.

**Supplementary Table 1. Clinical and pathological characteristics of HCC patients enrolled.**

Differential expression profiling of long non-coding RNA GAS5 and miR-126-3p in human cancer cells in response to sorafenib.

*Teresa Faranda, Ilaria Grossi, Michele Manganelli, Eleonora Marchina, Gianluca Baiocchi, Nazario Portolani, Marialuisa Crosatti, Giuseppina De Petro, Alessandro Salvi.*

| **CASE** | **GENDER** | **AGE** | **GRADING** | **BACKGROUND DISEASE** | **HBV** | **HCV** | **AFP** |
| --- | --- | --- | --- | --- | --- | --- | --- |
| 342 | M | 75 | G2-G3 | STEATOSIS | - | + | 4 |
| 343 | M | 64 | G2 | CHRONIC HEPATITIS | - | + | 1 |
| 345 | M | 72 | G2 | CHRONIC HEPATITIS | ~~-~~ | + | ~~-~~ |
| 347 | M | 63 | G2 | ACTIVE CIRRHOSIS | ~~-~~ | ~~-~~ | 8 |
| 348 | M | 60 | G3 | CHRONIC HEPATITIS | + | ~~-~~ | 10 |
| 349 | M | 71 | G2 | HEPATITIS | ~~-~~ | ~~-~~ | ~~-~~ |
| 350 | M | 78 | G3 | N/A | ~~-~~ | + | 3 |
| 351 | F | 69 | G2 | ACTIVE CIRRHOSIS | ~~-~~ | + | ~~-~~ |
| **352** | **M** | **82** | **G2** | **EARLY CIRRHOSIS** | **~~-~~** | **~~+~~** | **~~-~~** |
| 353 | M | 82 | G2 | NORMAL | ~~-~~ | ~~-~~ | 763 |
| 354 | M | 71 | G1 | STEATOSIS | ~~-~~ | ~~-~~ | ~~-~~ |
| **355** | **F** | **78** | **N/A** | **N/A** | **~~-~~** | **~~-~~** | **~~-~~** |
| **357** | **M** | **57** | **G3** | **CIRRHOSIS WITH STEATOSIS** | **+** | **~~-~~** | **17** |
| 359 | M | 75 | G2 | CIRRHOSIS | ~~-~~ | ~~+~~ | ~~-~~ |
| **360** | **M** | **71** | **G1** | **ACTIVE CIRRHOSIS** | **~~-~~** | **~~-~~** | **~~-~~** |
| 361 | M | 74 | G2 | EARLY CIRRHOSIS WITH MODERATE STEATOSIS | ~~-~~ | + | ~~-~~ |
| 362 | F | 79 | G2-G3 | CIRRHOSIS WITH STEATOSIS | ~~-~~ | + | 13 |
| 363 | M | 74 | G2 | CIRRHOSIS WITH STEATOSIS | ~~-~~ | ~~-~~ | ~~-~~ |
| 364 | F | 77 | G3 | CIRRHOSIS WITH STEATOSIS | ~~-~~ | + | 7 |
| 365 | M | 76 | G3 | VON MEYENBURG COMPLEX | ~~-~~ | ~~-~~ | N/A |
| 366 | M | 65 | G2-G3 | CIRRHOSIS WITH STEATOSIS | + | ~~-~~ | ~~-~~ |
| **367** | **M** | **73** | **G2** | **CHRONIC HEPATITIS** | **~~-~~** | **+** | **9** |
| 368 | M | 73 | G3 | CHRONIC HEPATITIS | + | ~~-~~ | 7 |
| 369 | M | 68 | G3 | CIRRHOSIS WITH STEATOSIS | ~~-~~ | ~~-~~ | ~~-~~ |
| 370 | M | 74 | G2 | CHRONIC HEPATITIS | ~~-~~ | + | - |
| 371^**^ | F | 77 | G2 | CIRRHOSIS WITH STEATOSIS | ~~-~~ | + | 2 |
| 372 | M | 72 | G2 | CHRONIC HEPATITIS EARLY CIRRHOSIS WITH STEATOSIS | ~~-~~ | + | 10 |
| 373 | M | 73 | G2 | EARLY STEATOSIS | ~~-~~ | - | 2 |
| 374 | F | 78 | G2 / G1 | CIRRHOSIS | ~~-~~ | + | 2 |
| 375 | M | 71 | G2 | CIRRHOSIS WITH STEATOSIS | ~~-~~ | + | N/A |

*In bold, peripheral blood only*

*** Solid biopsies only*

**Supplementary Table 2. Differential expression levels of lncRNAs in sorafenib-treated HA22T/VGH cells *vs* treated with 0.1% DMSO.**

Differential expression profiling of long non-coding RNA GAS5 and miR-126-3p in human cancer cells in response to sorafenib.

*Teresa Faranda, Ilaria Grossi, Michele Manganelli, Eleonora Marchina, Gianluca Baiocchi, Nazario Portolani, Marialuisa Crosatti, Giuseppina De Petro, Alessandro Salvi.*

|  | | | |
| --- | --- | --- | --- |
| **Gene symbol** | **Fold Change ^*^** | **Regulation^*^** | ***P*-value** |
| *bcyrn1* | 0.08 | DOWN | 0.005632 |
| *bdnf-as* | 0.45 | DOWN | 0.022118 |
| *bok-as1* | 0.46 | DOWN | 0.043302 |
| *cdkn2b-as1* | 0.24 | DOWN | 0.003393 |
| *disc2* | 0.25 | DOWN | 0.008117 |
| *fas-as1* | 1.57 | UP | 0.008938 |
| *ftx* | 0.11 | DOWN | 0.004019 |
| *gas5* | 2.69 | UP | 0.008294 |
| *gnas-as1* | 0.43 | DOWN | 0.015903 |
| *heih* | 2.16 | UP | 0.024813 |
| *hotair* | 0.66 | DOWN | 0.049147 |
| *hottip* | 0.28 | DOWN | 0.000035 |
| *hoxa11-as* | 0.43 | DOWN | 0.012825 |
| *hoxa-as2* | 0.09 | DOWN | 0.018223 |
| *ipw* | 0.60 | DOWN | 0.049269 |
| *lucat1* | 0.29 | DOWN | 0.045304 |
| *neat1* | 0.23 | DOWN | 0.009658 |
| *nron* | 0.24 | DOWN | 0.013959 |
| *oip5-as1* | 0.38 | DOWN | 0.010743 |
| *pcat1* | 0.15 | DOWN | 0.043572 |
| *terc* | 0.43 | DOWN | 0.006213 |
| *tmem161b-as1* | 0.20 | DOWN | 0.004161 |
| *tug1* | 0.24 | DOWN | 0.001951 |

* sorafenib-treated cells *vs* untreated cells

**Supplementary Table 3. Differential expression levels of miRNAs in sorafenib-treated HA22T/VGH cells *vs* treated with 0.1% DMSO.**

Differential expression profiling of long non-coding RNA GAS5 and miR-126-3p in human cancer cells in response to sorafenib.

*Teresa Faranda, Ilaria Grossi, Michele Manganelli, Eleonora Marchina, Gianluca Baiocchi, Nazario Portolani, Marialuisa Crosatti, Giuseppina De Petro, Alessandro Salvi.*

|  | | | |
| --- | --- | --- | --- |
| **miR** | **Fold**  **Change *** | **Regulation*** | ***P*-value** |
| hsa-miR-126-3p | 0.44 | DOWN | 0.021598 |
| hsa-miR-148a-3p | 0.75 | DOWN | 0.030670 |
| hsa-miR-16-5p | 0.82 | DOWN | 0.049102 |
| hsa-miR-27b-3p | 0.86 | DOWN | 0.015058 |
| hsa-miR-3183 | 3.35 | UP | 0.002017 |
| hsa-miR-3907 | 2.87 | UP | 0.022169 |
| hsa-miR-4454 | 0.89 | DOWN | 0.039167 |
| hsa-miR-4516 | 2.43 | UP | 0.029799 |

* sorafenib-treated cells *vs.* untreated cells
